# Climate Change: Believing and Seeing Implies Adapting

**DOI:** 10.1371/journal.pone.0050182

**Published:** 2012-11-21

**Authors:** Kristina Blennow, Johannes Persson, Margarida Tomé, Marc Hanewinkel

**Affiliations:** 1 Faculty of Landscape Planning, Horticulture, and Agricultural Science, Swedish University of Agricultural Sciences, Alnarp, Sweden; 2 Department of Philosophy, Lund University, Kungshuset, Lundagård, Lund, Sweden; 3 Centro de Estudos Florestais, Technical University of Lisbon, Tapada da Ajuda, Lisboa, Portugal; 4 Research Unit Forest Resources and Management, Swiss Federal Institute for Forest Snow and Landscape Research, Birmensdorf, Switzerland; 5 Institute of Forestry Economics, University of Freiburg, Freiburg, Germany; George Mason University/Krasnow Institute for Advanced Study, United States of America

## Abstract

Knowledge of factors that trigger human response to climate change is crucial for effective climate change policy communication. Climate change has been claimed to have low salience as a risk issue because it cannot be directly experienced. Still, personal factors such as strength of belief in local effects of climate change have been shown to correlate strongly with responses to climate change and there is a growing literature on the hypothesis that personal experience of climate change (and/or its effects) explains responses to climate change. Here we provide, using survey data from 845 private forest owners operating in a wide range of bio-climatic as well as economic-social-political structures in a latitudinal gradient across Europe, the first evidence that the personal strength of belief and perception of local effects of climate change, highly significantly explain human responses to climate change. A logistic regression model was fitted to the two variables, estimating expected probabilities ranging from 0.07 (SD ±0.01) to 0.81 (SD ±0.03) for self-reported adaptive measures taken. Adding socio-demographic variables improved the fit, estimating expected probabilities ranging from 0.022 (SD ±0.008) to 0.91 (SD ±0.02). We conclude that to explain and predict adaptation to climate change, the combination of personal experience and belief must be considered.

## Introduction

Knowledge of factors that trigger humans to respond to climate change is crucial for effective climate change policy communication. Climate change has been claimed to have low salience as a risk issue because it cannot be directly experienced. Being a statistical phenomenon (as climate is defined in terms of average weather) climate is not straightforwardly observable, for example see Moser and Ekstrom [1]. Some authors (e.g. Whitmarsh [2]) even claim that it is ‘buried’ in familiar natural processes, such as short-term weather fluctuations. Large random fluctuations of climate variables over time make it less probable that people accurately detect small climate trends. Still, personal factors such as strength of belief in local effects of climate change have been shown to correlate strongly with responses to climate change [3], [4] and there is a growing literature on the hypothesis that personal experience of climate change (and/or its effects) explains responses to climate change [5]–[7]. Only recently empirical data on personal experiences of climate change has started to be collected *cf*. [8], and until now the hypothesis has remained untested.

Forest owners are likely to be highly sensitive to climate change, and the forestry of specific areas provides exemplary local level arenas for adaptation to climate change *cf*. [9], since biological systems are exposed to and directly dependent on the climate. Thus, we designed a questionnaire study to assess the perceptions of, and responses to, climate change among private forest owners in Sweden, Germany and Portugal. The countries were chosen to represent a north–south gradient across Europe, covering forest owners operating in a wide range of bio-climatic conditions as well as economic–social–political structures. Here we provide the first test of the hypothesis that the variables personal strength of belief and perception of local effects of climate change, explain human responses to climate change.

## Methods

We designed a questionnaire study to assess the perceptions and behaviour in relation to climate change of 1,588 private forest owners in Sweden (Kronoberg County), Germany (Black Forest) and Portugal (Chamusca County). The questions asked whether the owners had adapted their forest management practices in response to climate change, their personal beliefs in local effects of climate change, whether they had experienced climate change (and/or its consequences), in addition to seeking socio-demographic information on their gender, year of birth, level of education, fraction of household income from forestry, and size of holding (Table 1). The questionnaires were accompanied by a cover letter explaining the objectives of the study and for what purpose the collected data will be used. The questionnaires were returned voluntarily by the respondents.

**Table 1:** Questions assessing respondents' perceptions and behaviour relating to climate change, and socio-demographic variables; possible responses to the questions; and percentage responses of respondents (or other summary statistics, where noted) who answered yes and no to the question *Have you adapted your forest management in response to climate change*? (n = 828).

Question	Response options	Have not adapted (n = 529)	Have adapted (n = 299)	Test statistics
1. Do you think that the climate is changing to such an extent that it will substantially affect your forest? (n = 826)	Yes, definitely	13.5%	55.2%	W = 35431.5, p<2.2e-16
	Yes, probably	38.5%	36.7%	
	Do not know	15.0%	4.0%	
	Probably not	30.5%	4.0%	
	Definitely not	2.5%	0.0%	
2. Have you experienced any extreme weather conditions that you interpret as caused by long-term, global climate change? (n = 813)	Yes, definitely	12.0%	51.5%	W = 31993, p<2.2e-16
	Yes, probably	17.9%	24.2%	
	Do not know	21.5%	8.9%	
	Probably not	42.5%	14.3%	
	Definitely not	6.1%	1.0%	
3. When were you born? (n = 820)	19__	Mean 1951, range 1921–1985	Mean 1955, range 1918–1983	t = −4.132, d.f. = 652.646, p = 4.07e-05
4. What is your gender? (n = 827)	Man	86.7%	91.2%	χ^2^ = 3.310, d.f. = 1, p = 0.0689
	Woman	13.3%	8.8%	
5. What education do you have? (n = 820)	Elementary school or equivalent	25.5%	14.7%	χ^2^ = 24.239, d.f. = 5, p = 1.95e-4
	High school or equivalent	12.8%	7.2%	
	Professional education or equivalent	38.7%	50.5%	
	University education or equivalent	18.3%	21.5%	
	Professional education or equivalent and University education or equivalent	4.7%	6.1%	
6. How large share of the household's income (during 2009) came from the management unit? (n = 792)	<5%	41.0%	30.6%	W = 44102.5, p<2.2e-16
	6–15%	20.1%	10.8%	
	16–25%	10.4%	18.3%	
	26–50%	9.4%	17.6%	
	51–75%	6.8%	10.4%	
	76–100%	12.2%	12.2%	
7. What is the size of your management unit? (n = 821)	Approximately _______ha	Median 49, range 0.90–8500 ha	Median 60, range 1.0–5500 ha	W = 598, p<2.2e-16

n =  Numbers of responses. Test statistics for Wilcoxon rank sum test (W), Student's t-test (t), and χ^2^-test (χ^2^). Mean, median and ranges calculated from raw data before imputation.

The research adheres to Swedish law regarding research involving human participants (Swedish Act 2003:460) and the handling of personal data (Swedish Act 1998:204). No further approvement by the authors' equivalent to the institutional review board (Etikprövningsnämnden) is needed. This has been confirmed by a representative of the Etikprövningsnämnden. Furthermore, had such vetting of the research been requested by the law, it can only be made before the research is carried out.

**Figure 1:**
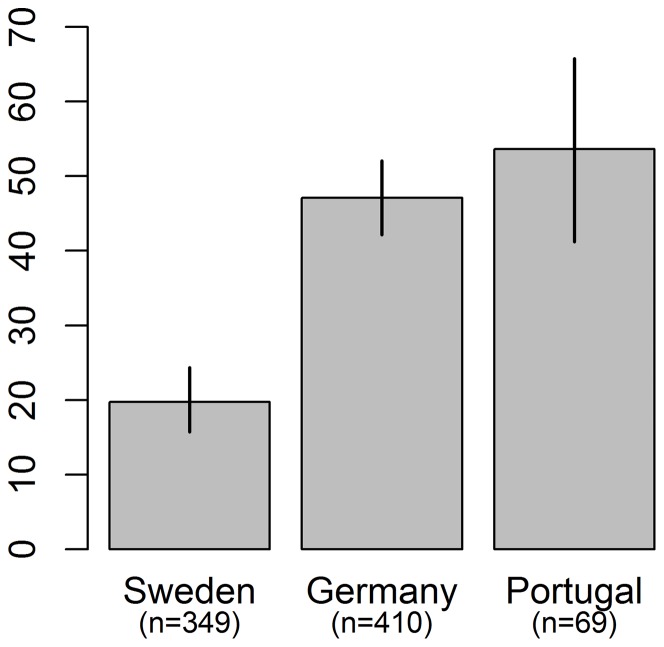
Adaptation of forest management to climate change by country. Bars denote 95% confidence intervals. Proportions of respondents (based on raw data before imputation) in Sweden who stated that they had adapted their management practices differed significantly to those in Germany (χ^2^ = 60.970, d.f. = 1, p = 5.80e-15) and Portugal (χ^2^ = 33.114, d.f. = 1, p = 8.69e-09).

**Figure 2:**
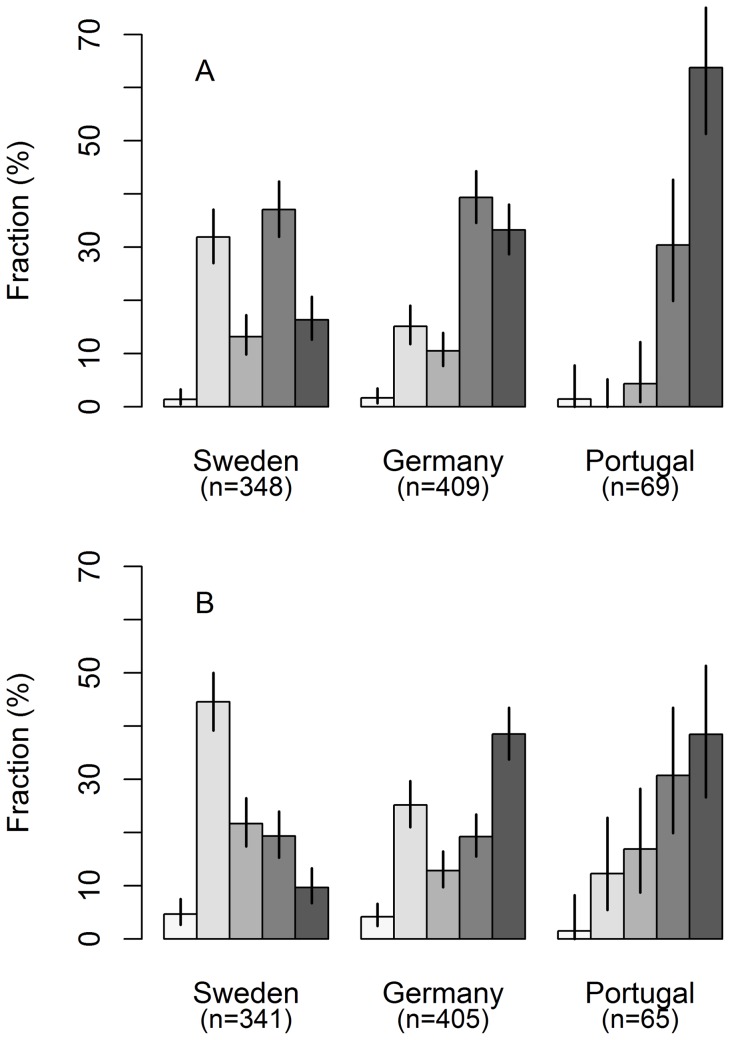
Respondents' perceptions of climate change. Respondents' alleged strength of belief in (A) local effects of climate change and (B) having experienced climate change, per country. The increasing shades of grey code for responses from “Definitely not” over “No, probably not”, “Do not know”, “Yes, probably”, to “Yes, definitely”, so that darker shades exhibit the strongest degree of belief in local effects of climate change (A) and having experienced climate change (B), respectively. Bars denote 95% confidence intervals per country. The strength of belief in local effects of climate change was significantly higher among respondents in Portugal than among respondents in Germany (W = 8899.5, p = 1.90e-07) and Sweden (W = 4778, p = 2.20e-16), and significantly higher in Germany than in Sweden (W = 52668, p = 1.09e-10). The strength of belief among respondents in having experienced climate change was significantly lower in Sweden than in Germany (W = 45853, p = 2.51e-16) and Portugal (W = 5553.5, p = 2.77e-11). The significance of differences (at α = 0.05) in strength of beliefs between countries was tested using the Wilcoxon rank sum test. Fractions refer to raw data before imputation.

The questionnaire was formulated in English and translated to the native language of respondents in each respective country. The Swedish forest owners were randomly sampled from contact persons with forest holdings larger than 5 ha listed in the Swedish Real Property Register (Swedish Act 2000:224). In Germany and Portugal the questionnaire was sent to all members of the forest owner organizations Forstkammer Baden-Württemberg and Associação dos agricultores de Charneca (in Chamusca), respectively. The questionnaires were distributed by mail during spring, 2010. A total of 871 forest owners returned the questionnaire (54.8%; Table S1). Details of the data collection procedure and quality control are described in Persson et al. [10].

**Figure 3:**
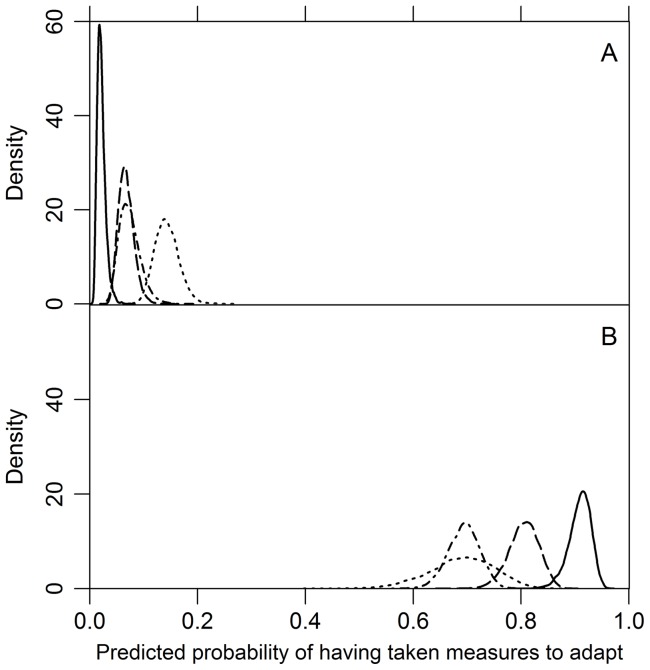
Simulated predicted probabilities of having taken measures to adapt for alternative models. Based on 10,000 simulations in each case, (A) the minimum and (B) maximum expected probability was 0.022 (SD ±0.008) and 0.91(SD ±0.02), respectively, for the model including both belief variables and socio-demographic variables (solid curve, Table 2), 0.07 (SD ±0.02) and 0.69 (SD ±0.03), respectively, for the model based on strength of belief in local effects of climate change only (dot-dashed curve, Table S2), 0.07 (SD ±0.01) and 0.81(SD ±0.03), respectively, for the model based on both belief variables (dashed curve, Table S3), and 0.14 (SD ±0.02) and 0.69 (SD ±0.06), respectively, for the model based on socio-demographic variables only with the year of birth held at its mean (dotted curve, Table S4).

To handle missing data (Table 1), we used the questions as variables to impute five complete data sets (n = 845) using maximum likelihood methodology. Statistical models based on relevant information from the observed portions of the dataset were used to construct multiple complete datasets where the imputations vary depending on the estimated uncertainty in predicting each missing value [11]. This methodology reduces bias and increases efficiency compared to listwise deletion [11]. We then applied logistic regression to all five datasets to explore and predict differences between groups differentiated by the two belief variables and socio-demographic variables, in stated adaptation of forest management to climate change. The best and most parsimonious models were chosen by backwards selection after adding all variables using Akaike's Information Criterion (AIC) as a performance indicator. To evaluate the fit of models with alternative specifications we used the likelihood ratio test and plots of receiver operating characteristics (ROC), using the area under the curves as a measure of concordance [12]. Expected probabilities of respondents having taken measures to adapt were estimated from 10,000 simulations drawn from the posterior distribution. All analyses were conducted using the R Project for Statistical Computing package v2.14.1 [13], particularly applying the libraries Amelia II for multiple imputation [14] and Zelig for logistic regression modeling [15], [16].

## Results

The largest fractions of respondents who stated they had taken measures to adapt the management of their forest to climate change were found in Portugal (53.6%) and Germany (47.1%), and the smallest fraction in Sweden (19.8%) (Fig. 1). The respondents' strength of belief in climate change (Question 1, Table 1) and having experienced climate change (Question 2) differed between countries. Portuguese respondents most strongly believed that the climate is changing to such an extent that it will substantially affect their forest and Swedish respondents least strongly (Fig. 2). The Swedish respondents also less strongly believed that they had experienced climate change than the German and Portuguese forest owners (Fig. 2).

A model based on stated strength of belief in local effects of climate change alone estimated expected probabilities of (self-reportedly) having taken measures to adapt the management of their forest to climate change ranging from 0.07 (SD ±0.02) to 0.69 (SD ±0.03) (Fig. 3, Table S2). Adding the explanatory variable stated strength of belief in having experienced climate change significantly improved the fit (χ^2^ = 63.808, d.f. = 1, p<0.0001) and estimated expected probabilities of having taken adaptive measures ranging from 0.07 (SD ±0.01) to 0.81 (SD ±0.03) (Fig. 3, Table S3).

**Table 2:** Diagnostic statistics of a model for predicting adaptive measures to climate change taken by forest owners based on personal belief variables and socio-demographic variables.

Variable	Value	Std. Error	t-stat	p-value
Intercept	0.810	0.257	3.153	1.64e-03
Country; (1 = Sweden, 0 otherwise)	−0.335	0.204	−1.641	0.101
S.b. climate change; (1 = Yes, probably, 0 otherwise)	−1.097	0.215	−5.107	3.57e-07
S.b. climate change; (1 = Do not know, 0 otherwise)	−2.069	0.360	−5.754	8.84e-09
S.b. climate change; (1 = No, probably not/Definitely not, 0 otherwise)	−2.816	0.359	−7.836	2.00e-14
S.b. exp. climate change; (1 = Yes, probably, 0 otherwise)	−0.623	0.249	−2.506	0.0122
S.b. exp. climate change; (1 = Do not know/Probably not/Definitely not, 0 otherwise)	−1.536	0.235	−6.524	9.12e-11
What education do you have; (1 = High, 0 otherwise)	0.476	0.211	2.258	0.0242
How large share of the household's income came from the forest management unit during 2009?;(1 = 16–75%, 0 otherwise)	1.040	0.201	5.184	2.53e-07

S.b. climate change, Strength of belief in local effects of climate change; S.b. exp. climate change, Strength of belief in having experienced climate change (and/or its consequences); High, Professional education or equivalent, and/or University education or equivalent. The model was fitted to five imputed datasets using logistic regression. Diagnostic statistics given for the logistic regression model include explanatory variables that are not significant at α = 0.05. The null deviance = 1105.649, the degrees of freedom for the null model = 844, residual deviance = 767.212, and the residual degrees of freedom = 836. The model fits the data significantly better than the null model (p<0.0001).

**Figure 4:**
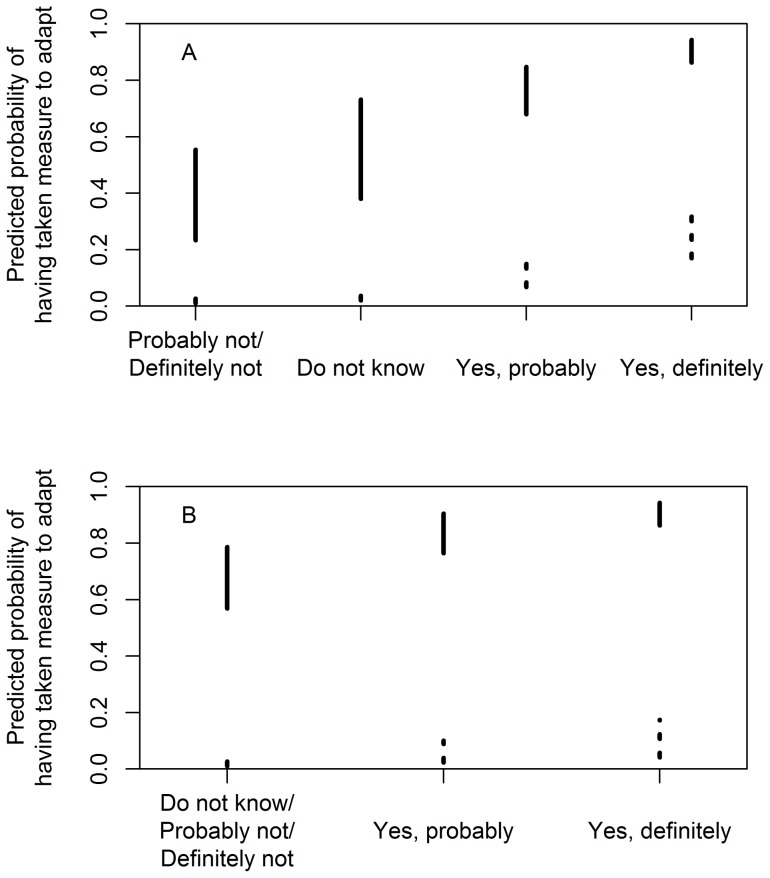
Sensitivity of the predicted probability of having taken measures to adapt to personal belief variables. Simulated 95% confidence intervals for the predicted probability were estimated using the model including both the two personal belief variables and socio-demographic variables (Table 2). Each confidence band was based on 10,000 simulations drawn while keeping all explanatory variables, except the variable (A) strength of belief in local effects of climate change and (B) strength of belief in having experienced climate change, at values contributing most strongly to high (solid lines) and low (dotted lines) probability of having taken measures to adapt, respectively. Confidence bands were simulated for all levels of the two personal beliefs variables used in the model, respectively.

**Figure 5:**
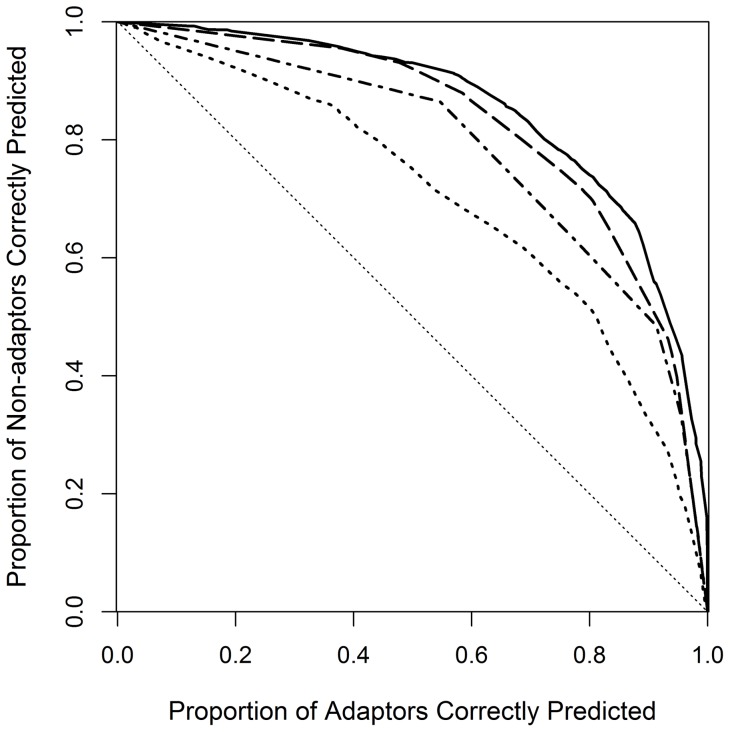
The predictive power of alternative models of adaptation of forest management to climate change. Receiver operating characteristics curves summarizing the predictive power of alternative models, showing changes in proportions of adaptors and non-adaptors correctly classified by each model as the threshold is varied. The area under curve (AUC) is: 0.852 for the model including both personal belief variables and socio-demographic variables (solid curve, Table 2); 0.778 for the model based on strength of belief in local effects of climate change alone (dot-dashed curve, Table S2); 0.824 for the model based on both personal belief variables (dashed curve, Table S3); and 0.700 for the model based on socio-demographic variables alone (dotted curve, Table S4). The diagonal thin dotted line represents the ROC curve that would have been obtained if probability values were selected randomly from a uniform distribution and unrelated to the data.

Both models fit the data better than a model based on socio-demographic variables (nationality, year of birth, level of education, and fraction of household income from forestry), which estimated expected probabilities ranging from 0.14 (SD ±0.02) to 0.69 (SD ±0.06) with year of birth held at its mean (χ^2^ = 123.260, DF = 3, p<0.0001 and χ^2^ = 187.068, DF = 4, p<0.0001, respectively) (Fig. 3, Table S4). The model with the best fit included both belief variables and the variables household financial dependency on forestry, level of education, and country in which the holding is located, estimating expected probabilities ranging from 0.022 (SD ±0.008) to 0.91 (SD ±0.02) for self-reported adaptive measures taken (Fig. 3, Fig. 4, Fig. 5) (Table 2). Neither the explanatory variables age (p = 0.972), gender (p = 0.402) and nationality (p = 0.101) of the respondent (Table 2), nor the (logarithm of the) size of the management unit (p = 0.593) contribute statistically significantly to this model at α = 0.05. This model fits the data significantly better than the model based only on the two personal belief variables (χ^2^ = 50.157, DF = 4, p<0.0001).

## Discussion

The models we present strongly suggest that the two variables reflecting personal climate change belief and perception highly accurately explain and predict adaptation, even for contrasting environments in a gradient across Europe (Fig. 3, Fig. 5) (Table 2). In contrast to Moser and Ekstrom [1] and Whitmarsh [2], our results clearly show that a substantial proportion (26.4%) of respondents strongly believe that they have directly perceived climate change (or events causally related to climate change) (Fig. 2). Widespread belief in having experienced climate change among citizens of the USA has also recently been reported [8]. Hence we suggest that it is not necessary to be able to perceive any (statistically defined) physical object in order to form strong beliefs that one has experienced climate change and adapt accordingly (cf. Rebetez [17]). While this tells us something about the prerequisites and efficacy of perception (of climate change) (see for example Weber [5], [6]), it does not necessarily tell us anything about the veracity of climate change *per se*. The general claim is not new. It is supported by numerous observations from other contexts. For a start, it is clear that we believe that we perceive many things that are not judged on reflection to be strictly observable – as causal relations (for example, see [18] and [19] or that lead melts at 327°C [20].

Our findings are pertinent to several common arguments and perspectives in the climate change adaptation literature.

First, our perceptions of climate change risk seem to exemplify a common pattern of overemphasizing the evidentiary value of recent perceptions related to the risk, for example see Weber [5]. Let us assume that adverse consequences of climate change are rare (as yet) in some regions of the world. As a result, perceptual evidence of the risk is often lacking. One implication would be that most people in these regions tend to underestimate climate change risks. Another implication would be that those who have recently experienced any of its rare consequences tend to exaggerate it. This is consistent with our results; a higher proportion of private Swedish forest owners believed in global warming in 2004 than after the cold winter in Sweden in 2010 (cf. Blennow & Persson [4] and Fig. 2).

The second perspective highlights the importance of our expectations for what we perceive, as suggested by Francis Bacon in the early 17th century [21] and confirmed by findings of modern psychological studies [22]. If we expect global warming then we will be prone to interpret what we ‘directly’ experience in accordance with this expectation. Accordingly, farmers who believe in climate change are reportedly more likely to distort their memories of past precipitation in the direction predicted by climate models [3].

Our study cannot discriminate between the hypothesis that direct experience causes belief in climate change and the hypothesis that the strength of belief in climate change explains direct experiences of climate change. Nor can it shed light on the role of other potential sources of learning on climate change (*cf*. [6] and [23]). What it does show is that both factors, the strength of belief in local effects of climate change and in having experienced climate change, have strong explanatory power and the two jointly accurately predict adaptation to climate change (Fig. 3, Fig. 4, Fig. 5).

Third, the relation we find is relevant in connection with contemporary models of adaptation and adaptive capacity. According to Lindner et al. [24] the adaptive capacity in European forestry has two components: the inherent capacity of trees and forest ecosystems and the socio-economic factors determining the ability to implement adaptation measures. They argue that adaptive capacity is much higher in northern Europe than in southern Europe. Our results suggest that adaptive capacity so defined and measured is not a sufficient condition for adaptation; the socio-demographic variables we have tested are much weaker correlated with adaptation than strength of belief in local climate change and experience of it (Table S4). Neither, it seems, does their model of adaptive capacity contain the necessary conditions for perceived adaptation. Our results show that it is (almost) sufficient to have the belief and experience (Fig. 3). Since our results also show that self-reported adaptation takes place in Portugal in southern Europe (Fig. 1), it seems that adaptive capacity has more or partly other components than Lindner et al. [24] report.

Fifty percent of the forest area in Europe is privately owned [25]. Hence, our results show that personal climate change belief and perception among those who make decisions for adaptation at the local level strongly influences the adaptive capacity of a substantial proportion of the European forest sector. Our conclusion conflicts with previous conclusions [25] in agreement with a general structural model [9], that the capacity to adapt to climate change in the European forest sector is largely dependent on the economic-social-political structures.

A model of proactive adaptation by Grothmann and Patt includes both social and cognitive variables [26]. According to their model, the perceived adaptive capacity has three subcomponents: perceived adaptation efficacy, perceived self-efficacy, and perceived adaptation costs. Neither of these components figures in our explanation – at least, not explicitly – and *vice versa*. Since the explanation we propose provides (nearly) sufficient conditions for adaptation (Fig. 3), in the circumstances we have studied, it seems to follow that the subcomponents proposed by Grothmann and Patt are not necessary conditions for perceived adaptation to climate change.

The two personal variables we identify are almost sufficient for explaining and predicting perceived adaptation (Fig. 3 and Table 2). But they do not occur in some of the most influential models of adaptation we have today. It seems unlikely that these models can explain the strong correlation we find. Hence, the contemporary models should not mistakenly be thought of as providing necessary conditions for perceived adaptation to climate change.

Further studies are needed to shed light on what shapes personal climate change beliefs. This includes testing the hypotheses that direct experience causes belief in climate change and that the strength of belief in climate change explains direct experiences of climate change, as well as revealing the role of other potential sources of learning on climate change (*cf*. [6] and [23]).

We conclude that measurements of two personal variables (strengths of belief in local effects of climate change and in having experienced climate change) are sufficient for accurately explaining and predicting whether or not European private forest owners will have taken measures to adapt to climate change (Fig. 5, Table 2), with expected probabilities ranging from 0.07 (SD ±0.01) to 0.81 (SD ±0.03) (Fig. 3). Our findings have implications for effective climate change policy communication, indicating that gathering and disseminating evidence of climate change and its effects could be an efficient strategy to increase peoples' perceptions of having experienced climate change (and hence to consider the need to take adaptive measures), at least among those who strongly believe in local effects of climate change.

## Supporting Information

Table S1 **Numbers of questionnaires distributed and returned per country.**
(DOC)

Table S2 **Diagnostic statistics of a model for predicting adaptive measures to climate change taken by forest owners based on strength of belief in local effects of climate change.**
(DOC)

Table S3 **Diagnostic statistics of a model for predicting adaptive measures to climate change taken by forest owners based on two personal belief variables: strengths of belief in local effects of climate change and having experienced climate change.**
(DOC)

Table S4 **Diagnostic statistics of a model for predicting adaptive measures to climate change taken by forest owners based on socio-demographic variables.**
(DOC)
