# Supplementary material for: Climate Change: Believing and Seeing Implies Adapting
Source: PLoS One. 2012 Nov 21;7(11):e50182. doi: 10.1371/journal.pone.0050182 (PMC3504002; doi:10.1371/journal.pone.0050182)
Supplement: Table S1 — Numbers of questionnaires distributed and returned per country. (DOC) [file pone.0050182.s001.doc]

**Table S1**. Numbers of questionnaires distributed and returned per country.

| ***Country*** | ***Distributed (n)*** | ***Returned (n)*** | ***Return ratio (%)*** |
| --- | --- | --- | --- |
| **Sweden** | 683 | 379 | 55.5 |
| **Germany** | 652 | 421 | 64.6 |
| **Portugal** | 253 | 71 | 28.0 |
| ***Total*** | *1588* | *871* | *54.8* |
