# Supplementary material for: Climate Change: Believing and Seeing Implies Adapting
Source: PLoS One. 2012 Nov 21;7(11):e50182. doi: 10.1371/journal.pone.0050182 (PMC3504002; doi:10.1371/journal.pone.0050182)
Supplement: Table S2 — Diagnostic statistics of a model for predicting adaptive measures to climate change taken by forest owners based on strength of belief in local effects of climate change. (DOC) [file pone.0050182.s002.doc]

**Table S2. Diagnostic statistics of a model for predicting adaptive measures to climate change taken by forest owners based on strength of belief in local effects of climate change.**

| **Variable** | **Value** | **Std. Error** | **t-stat** | **p-value** |
| --- | --- | --- | --- | --- |
| **Intercept** | 0.825 | 0.141 | 5.865 | 4.51e-09 |
| **S.b. climate change (1=Yes, probably, 0 otherwise)** | -1.431 | 0.184 | -7.780 | 7.46e-15 |
| **S.b. climate change (1=Do not know, 0 otherwise)** | -2.687 | 0.333 | -8.074 | 6.92e-16 |
| **S.b. climate change (1=No, probably not/Definitely not, 0 otherwise)** | -3.389 | 0.324 | -10.462 | 3.01e-24 |

S.b. climate change: Strength of belief in local effects of climate change.

The model was fitted to five imputed datasets using logistic regression. All diagnostic statistics given for the model are significant at α=0.05. The null deviance=1105.649, the degrees of freedom for the null model=838, residual deviance=881.176, and the residual degrees of freedom= 841. The model fits the data significantly better than the null model (p<0.0001).
