# Supplementary material for: Climate Change: Believing and Seeing Implies Adapting
Source: PLoS One. 2012 Nov 21;7(11):e50182. doi: 10.1371/journal.pone.0050182 (PMC3504002; doi:10.1371/journal.pone.0050182)
Supplement: Table S3 — Diagnostic statistics of a model for predicting adaptive measures to climate change taken by forest owners based on two personal belief variables: strengths of belief in local effects of climate change and having experienced climate change. (DOC) [file pone.0050182.s003.doc]

**Table S3. Diagnostic statistics of a model for predicting adaptive measures to climate change taken by forest owners based on two personal belief variables: strengths of belief in local effects of climate change and having experienced climate change.**

| **Variable** | **Value** | **Std. Error** | **t-stat** | **p-value** |
| --- | --- | --- | --- | --- |
| **Intercept** | 1.429 | 0.182 | 7.863 | 3.97e-15 |
| **S.b. climate change (1=Yes, probably, 0 otherwise)** | -1.017 | 0.202 | -5.028 | 5.02e-07 |
| **S.b. climate change (1=Do not know/No, probably not/Definitely not, 0 otherwise)** | -2.386 | 0.269 | -8.863 | 1.17e-18 |
| **S.b. exp. climate change (1=Yes, probably, 0 otherwise)** | -0.688 | 0.237 | -2.908 | 3.64e-03 |
| **S.b. exp. climate change (1=Do not know/Probably not/Definitely not, 0 otherwise)** | -1.691 | 0.222 | -7.604 | 7.85e-14 |

S.b. climate change, Strength of belief in local effects of climate change; S.b. exp. climate change, Strength of belief in having experienced climate change (and/or its consequences).

The model was fitted to five imputed datasets using logistic regression. All diagnostic statistics given for the model are significant at α=0.05. The null deviance=1105.649, the degrees of freedom for the null model=844, residual deviance=817.368, and the residual degrees of freedom= 840. The model fits the data significantly better than the null model (p<0.0001).
