# Supplementary material for: Climate Change: Believing and Seeing Implies Adapting
Source: PLoS One. 2012 Nov 21;7(11):e50182. doi: 10.1371/journal.pone.0050182 (PMC3504002; doi:10.1371/journal.pone.0050182)
Supplement: Table S4 — Diagnostic statistics of a model for predicting adaptive measures to climate change taken by forest owners based on socio-demographic variables. (DOC) [file pone.0050182.s004.doc]

**Table S4. Diagnostic statistics of a model for predicting adaptive measures to climate change taken by forest owners based on socio-demographic variables.**

| **Variable** | **Value** | **Std. Error** | **t-stat** | **p-value** |
| --- | --- | --- | --- | --- |
| **Intercept** | -18.656 | 12.359 | -1.509 | 0.131 |
| **Country (1=Sweden, 0 otherwise)** | -0.913 | 0.183 | -4.977 | 6.70e-07 |
| **Country (1=Portugal, 0 otherwise)** | -0.549 | 0.275 | 1.996 | 4.59e-02 |
| **When were you born?** | 0.00910 | 0.00632 | 1.439 | 0.150 |
| **What education do you have (1=High, 0 otherwise)** | 0.523 | 0.183 | 2.868 | 0.00415 |
| **How large share of the household’s income came from the forest management unit during 2009? (1=16-75%, 0 otherwise)** | 0.634 | 0.169 | 3.761 | 1.95e-04 |

High, Professional education or equivalent and/or University education or equivalent. The model was fitted using logistic regression to five imputed datasets. Diagnostic statistics shown include explanatory variables that are not significant at α=0.05. The null deviance=1105.649, the degrees of freedom for the null model=844, residual deviance=1004.436, and the residual degrees of freedom=839. The model fits the data significantly better than the null model (p<0.0001).
